# Baihu Jia Renshen Decoction may improve skeletal muscle and adipose tissue functions of type I diabetic rats by affecting pancreatic β-cell function

**DOI:** 10.1007/s13258-024-01607-6

**Published:** 2024-12-21

**Authors:** Shufang Chu, Deliang Liu, Hengxia Zhao, Ling Liu, Juntong Li, Gaoxiang Wang, Xuemei Liu, Huilin Li

**Affiliations:** 1 Department of Endocrinology, Shenzhen Traditional Chinese Medicine Hospital, No. 1, Futian District, Shenzhen, 518033 Guangdong China; 2 Department of Endocrinology, Shenzhen Traditional Chinese Medicine Hospital Affiliated to Nanjing University of Chinese Medicine, Shenzhen, 518033 China; 3 Department of Endocrinology, Jiangsu Province Hospital of Chinese Medicine, Affiliated Hospital of Nanjing University of Chinese Medicine, No. 155 Hanzhong Road, Qinhuai District, Nanjing, 210000 Jiangsu China

**Keywords:** Baihu Jia Renshen Decoction, Pancreatic β-cell, Skeletal muscle tissue, Adipose tissue, Type 1 diabetes mellitus

## Abstract

**Background:**

Baihu Jia Renshen Decoction (BJRD) is used for diabetes mellitus (DM) management in clinics.

**Objective:**

To elucidate the potential mechanism of BJRD in treating type 1 DM (T1DM).

**Methods:**

T1DM models were established via intraperitoneal injection of streptozotocin (STZ). Rats were subsequently randomly divided into the normal control (NC), model (MOD), insulin (INS), INS + BJRD-medium dose (MID), and INS + BJRD-high dose (HIGH) groups. The rats’ body weight was measured. Transcriptome sequencing was performed to detect differentially expressed genes (DEGs) in the muscle and adipose tissues. Quantitative real-time polymerase chain reaction was utilized to verify the DEG levels.

**Results:**

Body weights of MOD, INS, MID, and HIGH groups were significantly reduced as compared to those of NC group. Compared with NC group, MOD group showed significant Hspa1b and Notch3 downregulation and Camkk2 level elevation. Compared with MOD group, INS group showed further downregulation of the Hspa1b level, whereas MID group exhibited an increase. The Camkk2 levels in INS, MID, and HIGH groups were further reduced. The Notch3 levels did not significantly change in INS and MID groups, whereas that of HIGH group increased. Additionally, compared with NC group, MOD group demonstrated upregulation of the Myl1, Mylpf, Acacb, and Pygm levels and downregulation of Fasn level. Compared with MOD group, Myl1, Mylpf, and Pygm levels in INS, MID, and HIGH groups were down-regulated, whereas Fasn and Acacb levels were up-regulated.

**Conclusion:**

BJRD may influence pancreatic β-cell function, thereby enhancing the function of the skeletal muscle and adipose tissues in a T1DM rat model.

## Introduction

Type 1 diabetes mellitus (T1DM) is a distinct form of diabetes, marked by insulin deficiency and frequently diagnosed in the young population (Li et al. 2017). This autoimmune condition is driven by T lymphocytes, which specifically impair pancreatic β-cell function (Misra and Shukla 2023). Despite extensive research, the etiology of T1DM remains elusive, underscoring the disease’s complex pathogenesis (Blagov et al. 2023). Currently, insulin replacement therapy is the standard treatment for T1DM, albeit with notable limitations (Singh et al. 2023). Despite the considerable advances over the past decade, T1DM prevention and treatment remain suboptimal, with large and unexplained differences in the individual responses to interventions (Redondo and Morgan 2023). Consequently, exploring the treatment and pathogenesis of T1DM is crucial for enhancing DM management and reducing complications.

Zhang Zhongjing’s “Treatise on Febrile Diseases” documents a medicinal decoction, known as the Baihu Jia Renshen Decoction (BJRD), which can clear heat, invigorate Qi, and promote bodily fluid production (Xiong 2019). BJRD represents one of the earliest classical Chinese medicine formulations utilized in the treatment of DM. After years of clinical practice and extensive research, BJRD’s effectiveness has been scientifically proven and validated (Tian et al. 2020). BJRD is frequently employed in the clinical treatments for DM, and it has been shown to effectively improve the oxidative stress response of patients with DM, inhibit insulin resistance, enhance insulin sensitivity, and improve the curative effect (Yao et al. 2022; Zhou et al. 2022). Nonetheless, the extent of its influence and mechanism of action at varying doses remain unclear.

Owing to its unique advantages, transcriptome sequencing has attracted considerable attention in traditional Chinese medicine research, greatly advancing the field (Xin et al. 2017). RNA sequencing, also known as transcriptome sequencing, can unveil important insights into various genomic phenomena. This includes identifying gene fusion events, alternative splicing variants, mutations/deletions, and differential gene expressions. As a result, RNA sequencing offers a more comprehensive and detailed genetic map as compared with conventional DNA sequencing methods (Li and Wang 2021). Additionally, RNA sequencing has been used to conduct in-depth studies of the transcriptomes of different species, providing a better understanding of rare diseases and the taxonomic classification of various eukaryotes (Ergin et al. 2022). The present study aimed to investigate the body weight changes in streptozotocin (STZ)-induced T1DM rats following treatment with various dosages of BJRD, coupled with transcriptome sequencing, to unravel the underlying therapeutic mechanisms. Our study findings offer a novel strategy for T1DM prevention and treatment and elucidate the molecular underpinnings of traditional Chinese medicine in T1DM therapy.

## Materials and methods

### Animals

Prior to experimentation, the male Sprague Dawley rats (160–200 g) underwent an adaptive feeding period of 1 week to ensure that they were free from contamination or infection. The rats were provided with a standard diet and access to water. They were housed at room temperature (25 °C ± 5 °C) in a controlled environment with a 12-h light/dark cycle. Subsequently, they were randomly allocated into the following five groups, each containing six rats: normal control (NC) group (administered intraperitoneal (i.p) injection of 2 mL/kg citrate buffer (pH 4.5) and gavaged water); model (MOD) group (administered intraperitoneal (i.p) injection of 40 mg/kg STZ (Ahmed et al. 2023) and gavaged water); insulin (INS) group (administered STZ and treated with 8 IU/kg/d insulin by subcutaneous injection (Heidarisasan et al. 2018; Szilvássy et al. 2002)); INS + BJRD-medium dose (MID) group (administered STZ and treated with 8 IU/kg/d insulin by subcutaneous injection and gavaged 8.2 g/kg/d BJRD); and INS + BJRD-high dose (HIGH) group (administered STZ and treated with 8-IU/kg/d insulin by subcutaneous injection and gavaged 16.4 g/kg/d BJRD). After T1DM induction, the rats were treated with insulin and BJRD for 2 weeks. The formula for BJRD is as follows: gypsum, 5500 g; *Anemarrhena*, 1780 g; ginseng, 1100 g; licorice, 660 g; and Japonica rice, 990 g. The abovementioned decoction pieces were decocted twice with water, 10 times the amount of water each time, for 30 min, concentrated, cooled, pre-frozen in the refrigerator at − 80 °C overnight, and dried in a vacuum freeze dryer (the crude drug amount of the freeze-dried powder is 9.046 g of the decoction pieces per gram of the freeze-dried powder). The decoction pieces, including the ginseng tablets (batch number r0719116), raw gypsum (batch number s1417814), *Anemarrhena* (batch number z2319411), and licorice (batch number g0419515), were purchased from the traditional Chinese medicine decoction pieces’ factory of the Guangdong Medicinal Materials Company. Japonica rice is selected as the northeastern rice by COFCO Fulinmen. After treatment, all rats were weighed, fasted for 12 h, and anaesthetized by intraperitoneal injection of pentobarbital sodium (50 mg/kg). The tissue samples from the quadriceps muscle and epididymal adipose tissue were harvested and stored at − 80 °C. All animal experiments were approved by the Ethical Committee of the Guangzhou Forevergen Medical Laboratory Animal Center (approval number IACUC-AEWC-F2112017) and performed according to the IACUC handbook (third edition).

### Transcriptome sequencing

Transcriptome sequencing identified differentially expressed genes (DEGs) in the muscle and adipose tissues of each experimental group. PolyA mRNA purification was performed using Dynabeads Oligo(dT) via a hybridization technique. This step was followed by RNA fragmentation and double-stranded complementary DNA (cDNA) synthesis. Post-synthesis, the cDNA underwent end repair and A-tailing before ligation. The polymerase chain reaction (PCR) amplified the ligated cDNA, and library quality was assessed with an Agilent Bioanalyzer 2100 (Agilent Technologies, Santa Clara, CA, USA). We conducted RNA sequencing on the Illumina HiSeq 3000 platform. Data normalization and processing were executed using R software, applying quantile normalization. The DEGs were screened according to *P* of < 0.01, FDR of < 0.001, and|log_2_ ratio| of > 1.

### Gene ontology (GO) and kyoto encyclopedia of genes and genomes (KEGG) analyses

The intersection of DEGs in the muscle and adipose tissues of rats was analyzed by GO and KEGG. We identified the underlying biological processes of DEGs based on the GO (http://geneontology.org/page/go-enrichment-analyses) and KEGG pathways (http://www.genome.jp/kegg/pathway.html). The *P* value for each GO term was calculated using the right-sided hypergeometric tests. To correct for multiple tests, we utilized the Benjamini–Hochberg method (Al-Shahrour et al. 2008; Götz et al. 2008). The GO terms with a *P* of < 0.05 are considered significantly enriched.

### Quantitative real-time PCR (qRT-PCR)

The levels of DEGs Hspa1b, calcium/calmodulin-dependent protein kinase kinase 2 (Camkk2), Notch3, Myl1, Mylpf, fatty acid synthase (Fasn), acetyl-coenzyme A carboxylase beta (Acacb), and muscle-type glycogen phosphorylase (Pygm) were verified by qRT-PCR. TriQuick Reagent was used for total RNA extraction (R1100, Solarbio). Then, HiScript III RT SuperMix for qPCR (+ gDNA wiper) (R323-01, Vazyme) was utilized. ChamQ Universal SYBR qPCR Master Mix (Q711-02, Vazyme) was employed for qRT-PCR detection. The relative expression of the target genes was calculated using the 2^−ΔΔCt^ method. In the present study, GAPDH was employed as a reference gene. The primers utilized in this study are listed in Table 1.

**Table 1:** The primers used in the present study

Name	Sequences (5' → 3')
Hspa1b-F	GCTGCTGGTGCACGATTCTT
Hspa1b-R	TTCGCAGGAAGGAAACACCA
Camkk2-F	AAGAAGCTGATCCGACAGGC
Camkk2-R	TCAGGGGAGGTCCTGTATGG
Notch3-F	ACATCAATGAGTGCCGACCC
Notch3-R	GAAGTGCCCACCTGGATCTT
Myl1-F	TGGCACCAAAGAAAGACGTG
Myl1-R	TGCCTCCTTGAATTCCTCCT
Mylpf-F	GCCAGTGGGCCTATCAACTT
Mylpf-R	AGCAGCTCCTCCAAGAATTG
Fasn-F	TGTACCCTCTAGCTGGACCC
Fasn-R	CCAGGCTAAGGGCAATGGAA
Acacb-F	CTAGGTCACTGCTGCCACTC
Acacb-R	AGACCATTCAGACAACTGCGT
Pygm-F	CAGTGTTCGTGGCCTAGCTG
Pygm-R	CAGGTAGTAGATCCTCTTGGGGT
GAPDH-F	TCTCTGCTCCTCCCTGTTCT
GAPDH-R	CCGATACGGCCAAATCCGTT

### Statistical analysis

Statistical analysis was performed using GraphPad Prism 8.0 software. The measurement data were presented as mean ± standard deviation. The comparisons between multiple groups were made using one-way analysis of variance. *P* < 0.05 indicated statistical significance.

## Results

### Comparison of the body weight of rats among the different groups

**Fig. 1:**
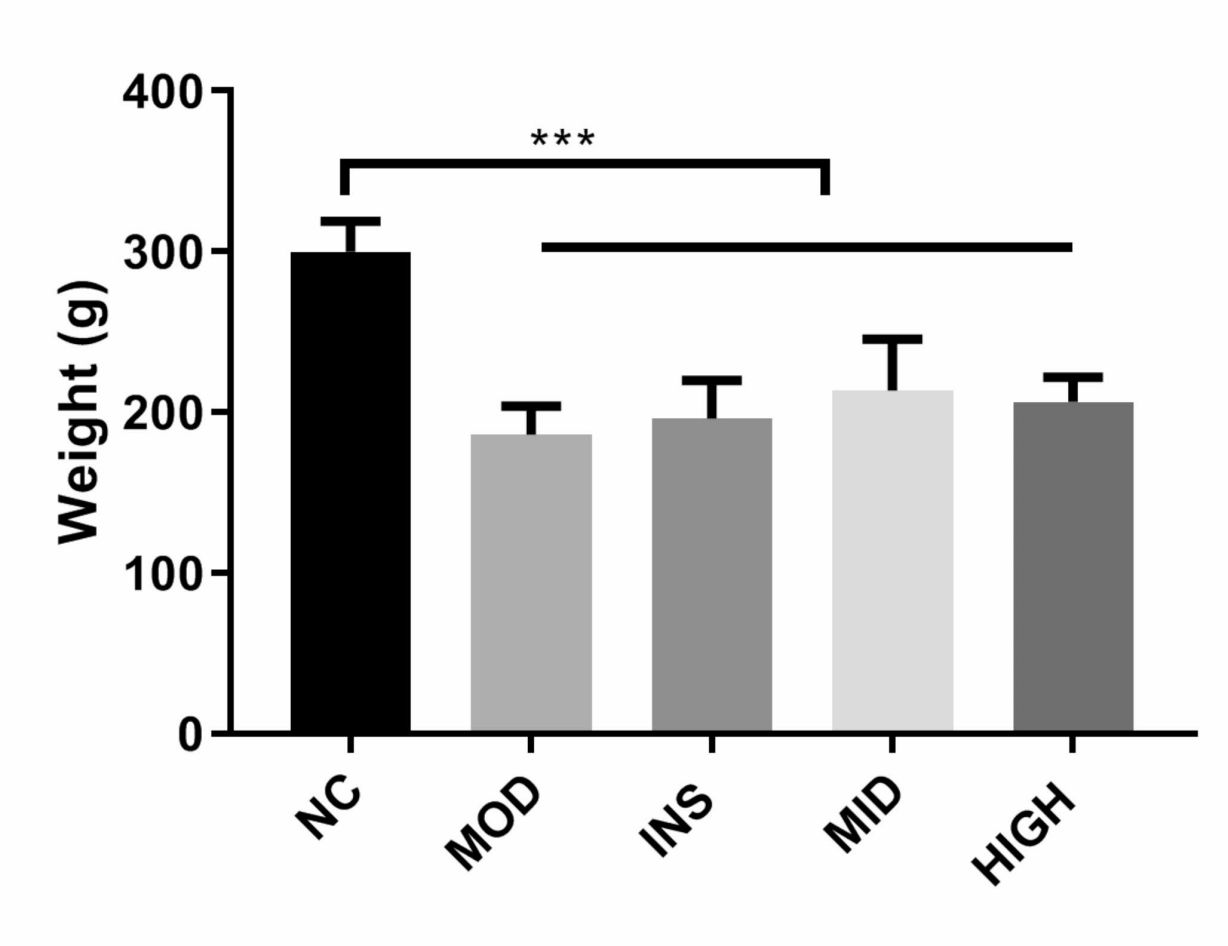
Comparison of the body weight of rats among the different groups. ***P < 0.001

First, we used STZ for T1DM induction; the, the rats were started with insulin and BJRD, which lasted for a fortnight. The body weights of rats in the MOD, INS, MID, and HIGH groups showed a significant decrease as compared with those of the NC group. The body weight of each treatment group (INS, MID, and HIGH groups) was slightly but not significantly elevated as compared with that of the MOD group (Fig. 1).

### DEGs in skeletal muscle tissue of rats in various groups and functional analysis

**Fig. 2:**
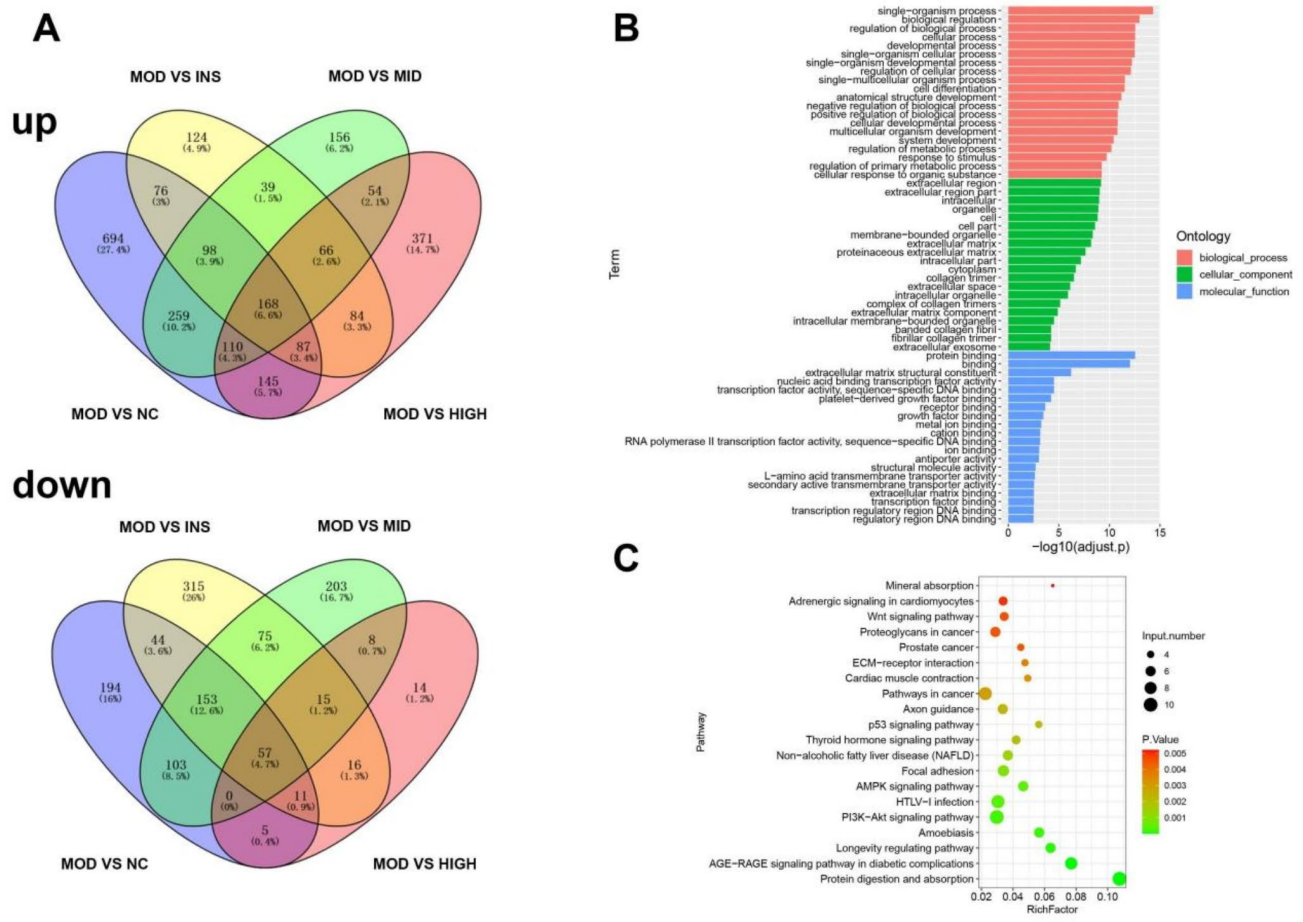
DEGs in rat skeletal muscle tissues across various groups, with functional analysis. **A** Transcriptome sequencing identified both up-regulated and down-regulated DEGs in the skeletal muscle tissue of rats from each group. **B**, **C** The intersection of DEGs in rat muscle tissue was analyzed using the GO and KEGG pathways. DEGs, differentially expressed genes; GO, Gene Ontology; KEGG, Kyoto Encyclopedia of Genes and Genomes

Next, we used transcriptome sequencing to analyze the DEGs in the skeletal muscle tissue of the various rat groups. In the skeletal muscle tissue of the MOD group, 1637 genes were up-regulated and 567 genes were down-regulated as compared with that of the NC group. Compared with the MOD group, the INS group showed 742 up-regulated and 686 down-regulated genes. In the MID group, 950 genes were up-regulated and 614 were down-regulated, whereas, in the HIGH group, there were 1,085 up-regulated and 126 down-regulated genes (Fig. 2A). Furthermore, we performed GO and KEGG analyses on the intersection of DEGs in the rat skeletal muscle tissues. The GO analysis showed that the DEGs in the rat skeletal muscle tissue were mainly concentrated in biological processes, including the single-organism process, biological regulation, biological process regulation, and cellular process; cellular component of the extracellular region, extracellular region part, intracellular, and organelle; molecular function of protein binding, binding, extracellular matrix structural constituent, and nucleic acid binding transcription factor activity (Fig. 2B). KEGG analysis showed that the DEGs in the rat skeletal muscle tissues were mainly enriched in protein digestion and absorption, PI3K-Akt signaling pathway, HTLV-I infection, and pathways in cancer, among others (Fig. 2C).

### Validation of the DEGs in the rat skeletal muscle tissue

**Fig. 3:**
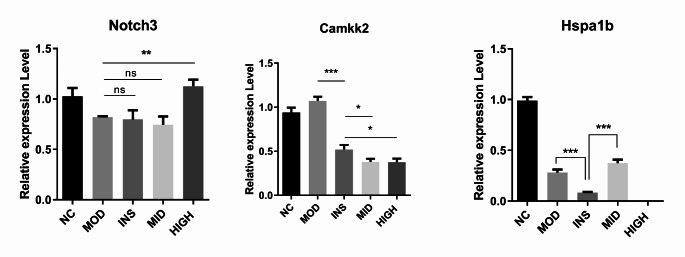
qRT-PCR verification of the expression of DEGs (Hspa1b, Camkk2, and Notch3) in rat skeletal muscle tissue. qRT-PCR, quantitative real-time polymerase chain reaction; DEGs, differentially expressed genes. ns (not significance), *P < 0.05, **P < 0.01, ***P < 0.001

We then validated the DEGs in the rat skeletal muscle tissues. Our literature review revealed that heat shock protein 70 (HSP70, Hspa1b), as a molecular chaperone, serves as a biomarker for cellular stress and plays a key role in maintaining cell homeostasis by preventing cell apoptosis and affecting energy metabolism, facilitating muscle adaptation processes, and interacting with other signaling pathways (Petersen and Shulman 2018). In T1DM patients with diabetic nephropathy, they also had elevated HSP 60 kDa isoform #A4, HSP71 kDa isoform #A30, and HSP27 kDa isoform #6 levels, but their HSP27 kDa isoforms #A90 and #A71 were decreased (Tessari et al. 2007). Additionally, T1DM induced by STZ injection increased the HSP90 levels in the heart but it decreased the HSP90 levels in the liver (Atalay et al. 2004). Calcium homeostasis is essential for maintaining pancreatic β-cell viability and function, and it plays a key role in preventing the development of DM. The disruption of calcium homeostasis will inactivate the Camkk2-AMP-activated protein kinase (AMPK) pathway (Yuan et al. 2018). Furthermore, the Notch3 receptor is a member of the mammalian Notch family receptors (Notch1–4) and plays a crucial role in regulating cell proliferation, differentiation, and apoptosis (Ye et al. 2013). Therefore, we measured the Hspa1b, Camkk2, and Notch3 levels. Compared with that of the NC group, the Hspa1b level was significantly down-regulated in the MOD group. Compared with the MOD group, the INS group showed a further down-regulated Hspa1b level, whereas, in the MID group, the Hspa1b level was increased, suggesting that BJRD plays a role in HSP70-related expression in rat skeletal muscle tissues. Additionally, the Camkk2 level was increased in the MOD group, as compared to that of the NC group. Compared with the MOD group, the INS, MID, and HIGH groups showed a further reduced Camkk2 level. Compared with the NC group, the MOD group exhibited a downregulation of the Notch3 level. In comparison to the MOD group, the INS and MID groups did not demonstrate a significant change in the Notch3 levels, whereas, in the HIGH group, the Notch3 level was increased (Fig. 3).

### DEGs in the adipose tissue of rats in various groups and functional analysis

**Fig. 4:**
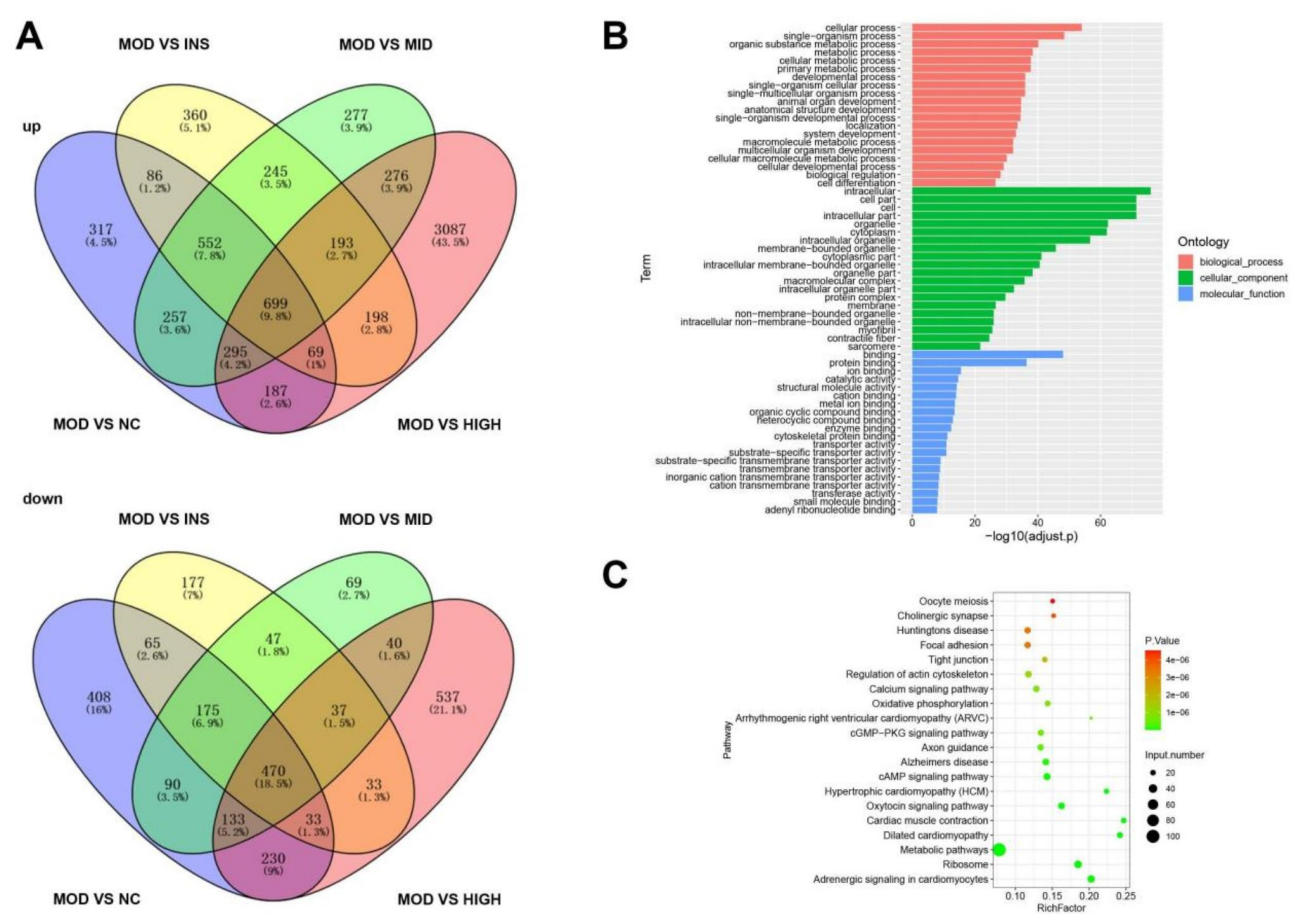
DEGs in rat adipose tissues across different groups, with functional analysis. **A** Transcriptome sequencing was conducted to identify both the up-regulated and down-regulated DEGs in the adipose tissues of rats from each group. **B**, **C** The intersection of DEGs in the rat adipose tissues was analyzed using the GO and KEGG pathways. DEGs, differentially expressed genes; GO, Gene Ontology; KEGG, Kyoto Encyclopedia of Genes and Genomes

We employed transcriptome sequencing to analyze the DEGs in the adipose tissue of each rat group. In the adipose tissue, the MOD group exhibited 2462 up-regulated genes and 1604 down-regulated genes as compared with the NC group. Relative to the MOD group, the INS group exhibited 2,402 up-regulated and 1,037 down-regulated genes, the MID group showed 2,794 up-regulated and 1,061 down-regulated genes, and the HIGH group demonstrated 5,004 up-regulated and 1,513 down-regulated genes (Fig. 4A). Furthermore, the intersection of the DEGs in the rat adipose tissue was analyzed by GO and KEGG. GO analysis revealed that the DEGs were mainly enriched in biological processes, including cellular, single-organism, organic substance metabolic, and metabolic processes; the cellular component of the intracellular region, cell part, cell, and intracellular part; and the molecular function of binding, protein binding, ion binding, and catalytic activity (Fig. 4B). KEGG analysis showed that the DEGs were primarily involved in the metabolic pathway, cAMP signaling pathway, HTLV-I infection, and cGMP-PKG signaling pathway (Fig. 4C).

### Validation of DEGs in the rat adipose tissues

**Fig. 5:**
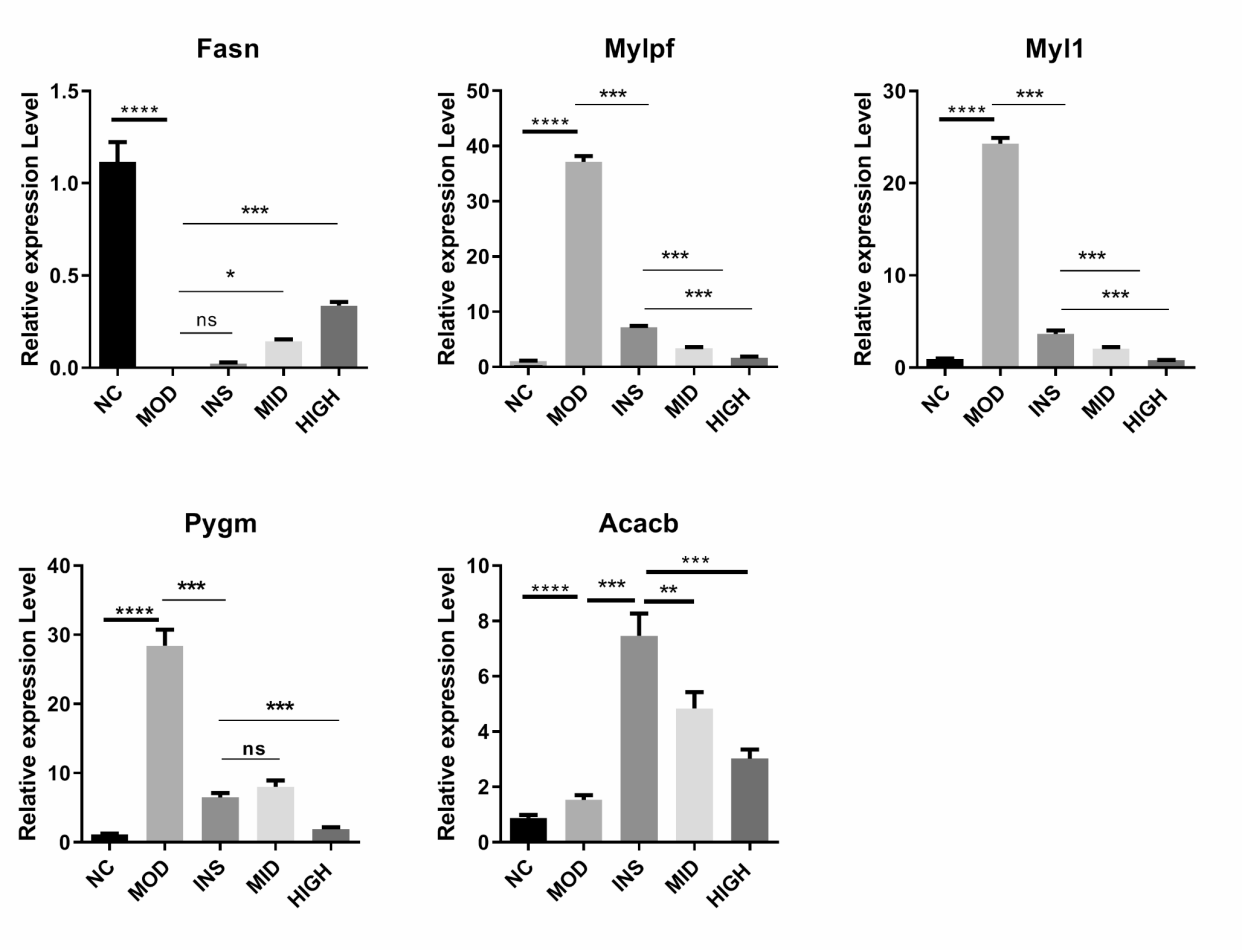
qRT-PCR verification of DEG (Myl1, Mylpf, Fasn, Acacb, and Pygm) expressions in rat adipose tissues. qRT-PCR, quantitative real-time polymerase chain reaction; DEGs, differentially expressed genes. ns (not significant), *P < 0.05, **P < 0.01, ***P < 0.001

Subsequently, we verified the DEGs in the rat adipose tissues. A review of the relevant literature revealed that DM attenuates bladder smooth muscle contraction and enhances myosin light chain (MLC) phosphorylation in rabbits (Su et al. 2004). The upregulation of the Fasn gene in adipose tissues is associated with visceral fat accumulation, impaired insulin sensitivity, circulating fasting insulin, and elevated IL-6, leptin, and RBP4 levels. These findings suggest that the adipogenic pathway plays a crucial role in the causal relationship between the consequences of excessive energy intake and obesity and type 2 DM (T2DM) development (Berndt et al. 2007). Acacb is the rate-limiting enzyme involved in fatty acid oxidation. In Acacb-knockout mice, sustained fatty acid oxidation results in increased insulin sensitivity (Ma et al. 2011). Pygm is mainly expressed in the muscle, and its high expression is associated with DM (Ugur et al. 2019). Its expression level is low in fat, but it is significantly increased in the adipose tissue of the diabetic model (Kedra et al. 1997). Therefore, we detected the expression of Myl1, Mylpf, Fasn, Acacb, and Pygm. Compared with the NC group, the MOD group showed up-regulated Myl1, Mylpf, Acacb, and Pygm levels, whereas the Fasn level was down-regulated. Moreover, the INS, MID, and HIGH groups showed down-regulated Myl1, Mylpf, and Pygm levels, whereas the Fasn and Acacb levels were up-regulated (Fig. 5).

## Discussion

T1DM is a chronic metabolic disease that poses a significant treatment burden (Benioudakis et al. 2023). Its primary etiology involves the immune-mediated destruction of pancreatic β-cell, with genetic and environmental factors triggering autoimmunity (Wang et al. 2023). Despite the advances in the treatments of DM since insulin’s discovery, the clinical needs of patients with T1DM remain unmet (Min and Bain 2023). Indeed, this underscores the urgent need to investigate novel therapies and gain a deeper understanding of the pathogenesis to enhance T1DM management. In the present study, we combined the transcriptome sequencing results to elucidate the potential mechanism of BJRD’s therapeutic effect. We discovered that BJRD affected the pancreatic β-cell function and improved the muscle and adipose tissue functions in T1DM rats. The present study is the first to report BJRD’s mechanism for improving muscle tissue and adipose tissue functions in T1DM rats via transcriptome sequencing, which is also the innovation of the present study.

BJRD has been identified as crucial in DM management. Yao et al. have noted that BJRD ameliorates T2DM in rats by affecting the gut microbiota, enhancing intestinal permeability, and inhibiting the TLR4/NF-κB mediated inflammatory response (Yao et al. 2022). Liu et al. found that BJRD treatment promotes AMPK phosphorylation and reduces liver lipid accumulation in db/db mice (Liu et al. 2015). Additionally, BJRD has demonstrated efficacy in preventing lipid and carbohydrate metabolism alterations in mice on a high-fat diet and in improving dietary obesity, hepatic steatosis, and insulin resistance. These studies imply BJRD’s therapeutic potential in metabolic disorders involving lipid and carbohydrate metabolism (Lu et al. 2020). In the present study, we first induced T1DM in rats with STZ and then treated them with insulin and various BJRD doses. Our study findings showed a significant body weight reduction in rats post-STZ modeling. Compared with the MOD group, each treatment group experienced a slight weight increase. We then integrated the transcriptome sequencing results to further investigate BJRD’s therapeutic mechanisms.

Skeletal muscle is a highly dynamic and adaptable tissue within the human body. It accounts for approximately 40% of the total body weight in humans and contains a considerable amount of protein, ranging from 50 to 75% of the total protein content (Frontera and Ochala 2015). It serves as a primary model for studying disease mechanisms and evaluating the effects of various interventions (Lindholm et al. 2014). The skeletal muscle exhibits considerable plasticity under both physiological and pathological conditions (Lin et al. 2022). In the present study, we analyzed the DEGs of the skeletal muscle tissue in each group of rats by transcriptome sequencing and performed functional analysis. Our results indicate that the DEGs in rat skeletal muscle tissues are predominantly related to protein digestion and absorption, PI3K–Akt signaling pathway, HTLV-I infection, and pathways in cancer, among others. Future studies should delve deeper into the mechanisms of these enrichment pathways.

The onset of T1DM is characterized by an inflammatory environment that intensifies cellular stress responses, leading to disease manifestations (Burkart et al. 2008). Research in T2DM patients has established that the expression of the 72-kDa heat shock protein in the skeletal muscle is highly correlated with the metabolic status of patients with DM (Korányi et al. 2004). Heat pretreatment and induction of HSP70 could attenuate STZ-induced changes in hepatic carbohydrate metabolism and oxidative state (Gerazova-Efremova et al. 2019). Rasouli et al. confirmed that carbenoxolone may be induced by HSP70 and subsequently increase the IFN-γ levels, leading to the inhibition of IL-10 production in DM mice, producing toxic effects on the pancreatic β-cells, thereby worsening the disease (Rasouli et al. 2018). Our study revealed a significant downregulation of Hspa1b in the MOD group as compared with the NC group. Compared with the MOD group, the INS group showed further downregulation of the Hspa1b level, whereas that of the MID group was increased, suggesting that BJRD plays a role in HSP70-related expression in rat skeletal muscle tissues.

Camkk2 is a pivotal regulator of various physiological processes, including nutrient intake, glucose metabolism, insulin production, and fat formation. Its integral role in these mechanisms underscores its importance as a key signaling molecule in metabolic homeostasis (Williams and Sankar 2019). The loss of Camkk2 has been shown to protect mice from obesity, insulin resistance, and glucose intolerance induced by high-fat diets (Anderson et al. 2008). Ying et al. have reported that fibroblast growth factor 21 ameliorates diabetic aortic endothelial dysfunction in mice by activating the Camkk2/AMPKα pathway (Ying et al. 2019). Moreover, Zhong et al. (Zhong et al. 2023) demonstrated diosgenin’s efficacy in improving type 2 diabetic nephropathy by enhancing autophagy and mitochondrial dynamics via Camkk2. These studies suggest that Camkk2 plays a vital role in the progression of DM. Notch3 is a key driver of diabetic vascular disease (Wimmer et al. 2019). According to Anastasi et al., Notch3-mediated events have a regulatory effect on T-regulatory cell expansion and function. This modulation ultimately leads to protection against experimental autoimmune DM. The present study suggests that targeting the Notch pathway could potentially serve as a therapeutic intervention for T1DM (Anastasi et al. 2003). In the present study, the Camkk2 level was increased and the Notch3 level was down-regulated in the MOD group as compared with the NC group. Compared with the MOD group, the Camkk2 levels in the INS, MID, and HIGH groups were further down-regulated. Moreover, the Notch3 levels in the INS and MID groups were not significantly changed, whereas that of the HIGH group was increased. Our results confirmed that BJRD affected the expression of DEGs Hspa1b, Camkk2, and Notch3 in the skeletal muscle tissue of T1DM rats.

The adipose tissue demonstrates remarkable plasticity under both physiological and pathophysiological conditions, altering in size and cell composition (Maniyadath et al. 2023). It serves as a crucial metabolic regulator, involved in energy storage and dissipation, and bioactive molecule secretion (Tsuji and Tseng 2023). Disruptions in the biological processes of adipose tissues can lead to systemic physiological imbalances, contributing to metabolic disorders, such as obesity and T2DM (Auger and Kajimura 2023). In our study, we analyzed the DEGs in rat adipose tissues through transcriptome sequencing and conducted functional analyses. We found that the DEGs in the rat adipose tissues were mainly enriched in the metabolic pathway, cAMP signaling pathway, HTLV-I infection, and cGMP–PKG signaling pathway, among others. In the future, we plan to further explore the mechanisms of these enrichment pathways.

Serum MLC kinase is associated with T2DM, providing a new biomarker for the diagnosis of T2DM and its complications (Di et al. 2015). Additionally, high-fat diets cause the fiber type in male mice to switch from slow/oxidizing to fast/glycolytic (Myh7, Myl2, and Myl3 decreased; Myh2, Mylpf, Mybpc2, and Myl1 increased) (Moriggi et al. 2021). In line with previous studies, our study found an upregulation of Myl1 and Mylpf in the MOD group as compared with the NC group. This upregulation was reversed in the INS, MID, and HIGH groups. Fasn, a key enzyme in liver DNL, is associated with insulin resistance (Matsukawa et al. 2023). Xie et al. have reported that METTL3 diminishes liver insulin sensitivity and augments fatty acid metabolism through n6 methylation of Fasn mRNA (Xie et al. 2019). Through a protein–protein interaction analysis, Ge et al. found that Fasn and Camk2b were involved in DM pathogenesis (Ge et al. 2020). The Acacb gene plays an important role in fatty acid oxidation, and its alteration may influence susceptibility to type 2 diabetic nephropathy (Riancho et al. 2011; Tang et al. 2010). In STZ-induced DM mice, the excretion of urinary albumin was considerably increased in Acacb transgenic mice as compared with that of wild-type mice. The overexpression of Acacb could aggravate STZ-induced injury of renal podocytes in DM mice (Tanaka et al. 2018). Additionally, Pygm has been reported to be greatly elevated in the adipose tissue of DM models (Kedra et al. 1997). In the present study, we found that, as compared with the NC group, Myl1, Mylpf, Acacb, and Pygm levels were up-regulated in the MOD group, whereas the Fasn levels were down-regulated. Compared with the MOD group, the Myl1, Mylpf, and Pygm levels in the INS, MID, and HIGH groups were down-regulated, whereas the Fasn and Acacb levels were up-regulated. Our study confirmed that BJRD affected the expression of DEGs Myl1, Mylpf, Fasn, Acacb, and Pygm in the adipose tissues of T1DM rats.

## Conclusion

Our study data suggest that BJRD influences pancreatic β-cell function, thereby enhancing the functionality of skeletal muscle and adipose tissues in T1DM rats. Transcriptome sequencing was performed to identify the DEGs in the muscle and adipose tissues of rats and to analyze their functions. Subsequent verification confirmed that BJRD modulates the DEGs in both the muscle and adipose tissues of rats. Our study findings provide insights into the molecular mechanisms underlying BJRD’s therapeutic effects on T1DM, offering novel perspectives for the treatment of this condition.

## Data Availability

Data included in the manuscript.
